# Expression profiling with RNA from formalin-fixed, paraffin-embedded material

**DOI:** 10.1186/1755-8794-1-9

**Published:** 2008-04-19

**Authors:** Andrea Oberli, Vlad Popovici, Mauro Delorenzi, Anna Baltzer, Janine Antonov, Sybille Matthey, Stefan Aebi, Hans Jörg Altermatt, Rolf Jaggi

**Affiliations:** 1 Department of Clinical Research, University of Bern, Murtenstrasse 35 CH-3010 Bern, Switzerland; 2 Swiss Institute of Bioinformatics (SIB), CH-1015 Lausanne, Switzerland; 3 National Center of Competence in Research (NCCR) Molecular Oncology, Swiss Institute for Experimental Cancer Research (ISREC), Epalinges, Switzerland; 4 Pathology Länggasse, Forstweg 56, CH-3012 Bern, Switzerland

## Abstract

**Background:**

Molecular characterization of breast and other cancers by gene expression profiling has corroborated existing classifications and revealed novel subtypes. Most profiling studies are based on fresh frozen (FF) tumor material which is available only for a limited number of samples while thousands of tumor samples exist as formalin-fixed, paraffin-embedded (FFPE) blocks. Unfortunately, RNA derived of FFPE material is fragmented and chemically modified impairing expression measurements by standard procedures. Robust protocols for isolation of RNA from FFPE material suitable for stable and reproducible measurement of gene expression (e.g. by quantitative reverse transcriptase PCR, QPCR) remain a major challenge.

**Results:**

We present a simple procedure for RNA isolation from FFPE material of diagnostic samples. The RNA is suitable for expression measurement by QPCR when used in combination with an optimized cDNA synthesis protocol and TaqMan assays specific for short amplicons. The FFPE derived RNA was compared to intact RNA isolated from the same tumors. Preliminary scores were computed from genes related to the ER response, HER2 signaling and proliferation. Correlation coefficients between intact and partially fragmented RNA from FFPE material were 0.83 to 0.97.

**Conclusion:**

We developed a simple and robust method for isolating RNA from FFPE material. The RNA can be used for gene expression profiling. Expression measurements from several genes can be combined to robust scores representing the hormonal or the proliferation status of the tumor.

## Background

Breast cancer has been widely studied in the past and molecular characterization has increased the understanding of biological pathways that are altered during neoplastic transformation of cells [1-4]. However, the findings based on molecular profiling have not yet altered diagnosis, and decisions about treatment still rely mostly on histopathological and immunohistochemical techniques which are at best semi-quantitative [5,6]. Currently, many patients with primary, non-metastatic breast cancer with positive estrogen receptor (ER) status undergo several cycles of chemotherapy, although a substantial proportion of them does not benefit from it. Presently, no conventional parameters exist for many patients which allow to identify individuals who will benefit from chemotherapy. Personalized diagnosis on the basis of highly specific molecular analyses has the potential to improve the situation of many patients by optimizing medication, and at the same time, sparing others from unnecessary treatment regimens.

DNA chip studies are based on measuring gene expression for many genes in parallel [1,4,7,8]. Most protocols for gene expression analysis on the basis of DNA chips are sensitive to RNA degradation and RNA must be isolated from freshly prepared or FF tumor material. As a consequence, material is fairly limited and often originates from convenience samples of heterogeneous patients. Many of these studies including meta-analyses have revealed genes and biological functions of their products which are relevant for classification and prognosis [9,10]. However, many samples were derived from patients who did not participate in clinical studies and their treatment regimens were not standardized. Therefore, follow up data must still be interpreted with caution.

Obviously, procedures based on formalin-fixed, paraffin-embedded (FFPE) material would greatly facilitate and speed up research in this area as large amounts of highly valuable material and clinical data have already been collected. In many cases, FFPE blocks are still available and they could be used for a molecular analysis. Especially material from clinical trials would allow investigating distinct clinical questions with existing material rather than material from newly designed studies.

Many efforts are currently made to individualize diagnosis of breast cancer by including molecular parameters into diagnosis. Fresh frozen material would obviously be ideal for a molecular analysis by gene expression measurements but it may be difficult to implement novel procedures which complicate current workflows of daily routine. Procedures based on FFPE material would be more feasible as they do not interfere with current protocols and they do not affect routine diagnosis as material for molecular analysis could be collected after standard diagnosis has been terminated. Only relatively few molecular approaches have been described which are based on FFPE material. For example, Paik and co-workers have established a recurrence score (RS, Oncotype DX), it allows to quantify the likelihood of distant recurrence and to predict the magnitude of chemotherapy benefit [11,12].

It is generally accepted that molecular profiles which reflect primarily biological characteristics of tumor cells, may complement clinical and histopathological diagnosis, resulting in a more detailed characterization of individual tumors, a pre-requisite for better treatment decisions. In this study we present the development of a novel procedure for RNA isolation from FFPE material and an optimized workflow for expression measurements by QPCR.

## Methods

### Human breast cancer samples

Human breast cancer specimens were divided into two aliquots, one of which was processed for histological diagnosis by fixation with formalin and embedding in paraffin. FFPE material was obtained from the Institute of Pathology (University of Bern) and the Pathology Länggasse, Bern. Tissue (3–5 mm thick slices of tumor) was fixed over night in buffered formalin and processed for paraffin embedding in a Tissue Processing Center TPC 15 (Medite Medizintechnik, Germany). The second aliquot was frozen on dry ice and stored at -80°C. Fresh frozen material was obtained from the Tumorbank Bern. Both, FF and FFPE samples were checked by hematoxylin and eosin staining and only samples with more than 50% tumor cells were used for this study. An informed consent to use the material for research was obtained from all the patients.

### RNA Extraction

Intact RNA was isolated from four 25 μm thick kryo-sections of approximately 0.5 cm^2^. The tissue was homogenized in 420 μl lysis buffer (4 M guanidinium thiocyanate, 30 mM Tris pH 8.0, 1% Triton-X-100), 8.0,1 using a TissueLyser (Mixer Mill, Retsch GmbH, Haan, Germany) at 15 Hz for 3 min. Total RNA was bound to silica-based columns (Epoch Biolabs, Huston Texas), treated with DNase I (30 Kunitz units for 20 min. at room temperature; Qiagen, Hilden, Germany), washed once with lysis buffer (containing 30% ethanol) and once with 20 mM NaCl (containing 20% ethanol) and eluted in 50 μl 10 mM Tris pH 7.4, 0.1 mM EDTA and stored at -20°C. RNA quantity was measured on an ND-1000 spectrophotometer (NanoDrop Technologies, Wilmington, DE) and quality assessed by capillary electrophoresis with an Agilent 2100 Bioanalyzer (Agilent Technologies, Inc., Santa Clara, CA) using Agilent RNA 6000 Series Nano kits.

**Figure 1:**
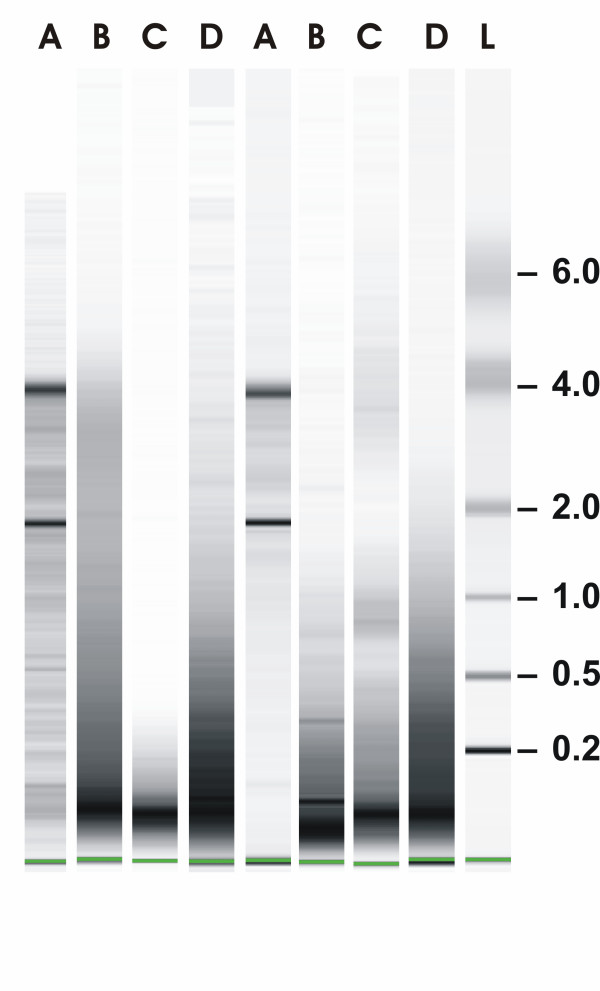
**RNA isolation and characterization**. Total RNA was isolated from kryo-sections (lanes A) and from paraffin sections according to the RNeasy FFPE protocol of Qiagen (lanes B), the ncLysis protocol of Applied Biosystems (lanes C) or according to our own protocol (lanes D). Aliquots of each RNA were separated by capillary electrophoresis (Agilent Bioanalyzer) on Nano chips along with RNA ladder (L; Ambion). Shown are RNAs from two representative tumors (Tu#10 and #18).

RNA was isolated from ten 10 μm thick FFPE sections according to the RNeasy FFPE protocol of Qiagen (Fig. 1, lanes B), the ncLysis protocol of Applied Biosystems (lanes C) and the protocol developed in our laboratory (lanes D). Paraffin sections were de-paraffinized with xylene, washed with ethanol and dried in a speed vac. For our own protocol, 200 μl lysis buffer (4 M guanidinium thiocyanate, 30 mM Tris, pH 8.0, 1% Triton-X-100) was added to the dried sections and immediately homogenized in a Mixer Mill at 20 Hz for 4 min. Proteinase K (Roche Diagnostics, Mannheim, Germany) was added (1 mg/ml final concentration) and tissue was digested for 1 hour at 55°C. One milliliter dilution buffer (30 mM Tris, pH 8.0, 1% Triton-X-100) was added to each lysate and digestion continued for 1 hr after adding fresh proteinase K (final concentration 1 mg/ml). RNA was de-modified by adding 318 μl of de-modification solution (5 M NH_4_Cl) and incubating at 94°C for 20 min or as described in the text. RNA was bound to silica-based columns and digested with DNase I as described for fresh-frozen tissue samples. The reproducibility of our own procedure was tested by isolating several independent RNAs from consecutive sections of the same tissue block. About 10 μg of total RNA could be isolated from 5 to 10 FFPE sections (0.5–1 cm^2^/section). RNA was isolated from closely matched sections using the RNeasy FFPE kit (Qiagen) or the ncLysis system (Applied Biosystems) according to the protocols included with the kits. In both cases, the RNA was purified on silica-based columns. 22 samples were available. In 14 cases sufficient RNA was obtained from all 4 parallel isolations. In 2 cases of FF material (samples 4 and 11) and in 6 cases of FFPE material (samples 1, 5, 7, 9, 12 and 21) less than 1.5 μg RNA could be isolated with the ncLysis protocol. These samples were excluded from further analysis.

### cDNA synthesis and QPCR

Aliquots of 100 to 500 ng of total RNA were reverse transcribed using MultiScribe™ MuLV reverse transcriptase (High-Capacity cDNA Archive Kit; Applied Biosystems, Foster City, CA, USA) and random or gene-specific primers. Reverse primers were kindly provided by Applied Biosystems, they were used at 1 μM each, cDNAs were made in the presence of 3, 10 or 22 reverse primers as 3-plex, 10-plex or 22-plex, respectively. Regular Assays on Demand (Applied Biosystems) were used for QPCR (Table 1). Manually designed assays coding for short, medium-size and long amplicons of the insulin growth factor-binding protein 5 (IGBP5) were selected with Primer Express (Version 3, Applied Biosystems). Forward primer and probe were kept constant for all assays while reverse primers were selected such that amplicons of different sizes were generated [13]. QPCR reactions were carried out in triplicates in a final volume of 10 μl in 1× FAST Master mix (Applied Biosystems) and cDNA corresponding to 4 ng total RNA. QPCR was performed on an ABI 7500 FAST instrument (2 min at 95°C, followed by 45 cycles of 95°C for 3 sec and 60°C for 30 sec). The quality of the assays and the absence of contaminating DNA were assessed with water and RNA instead of cDNA, respectively (data not shown). Three positive controls containing cDNA derived of ZR-7-51 cells were included on each 96-well plate. Cycle threshold values (Ct) were determined using the SDS software of the 7500 FAST System (Version 1.3.1). Constant threshold values were set for each gene throughout the study.

**Table 1:** QPCR assays. QPCR assays (Assays on Demand) were from Applied Biosystems (Palo Alto, CA). Reverse primers from each assay were used for the synthesis of gene-specific cDNAs. They were provided separately by Applied Biosystems. Three assays (IGBP5_short, IGBP5_medium, IGBP5_long) were designed manually.

**AoD**	**Assay**	**Acc_Nr**	**AmpliconSize**	**Module**
Hs00608023_m1	BCL2	NM_000633	81	Estrogen
Hs00221277_m1	CEGP1	NM_020974	64	Estrogen
Hs00174860_m1	ESR1	NM_000125	62	Estrogen
Hs00172183_m1	PGR	NM_000926	118	Estrogen
Hs00180450_m1	GRB7	NM_005310	70	Her2
Hs01001598_g1	HER2	NM_004448	55	Her2
Hs00952036_m1	CTSL2	NM_001333	72	Invasion
Hs00171829_m1	STMY3	NM_005940	66	Invasion
Hs01030097_m1	CCNB1	NM_031966	66	Proliferation
Hs01032443_m1	MKI67	NM_002417	66	Proliferation
Hs00231158_m1	MYBL2	NM_002466	81	Proliferation
Hs00269212_m1	STK15	NM_003600	85	Proliferation
Hs00153353_m1	SURV	NM_001168	93	Proliferation
Hs99999903_m1	ACTB	NM_001101	171	Reference
Hs00266705_g1	GAPDH	NM_002046	74	Reference
Hs99999908_m1	GUSB	NM_000181	81	Reference
Hs99999902_m1	RPLP0	NM_001002	105	Reference
Hs00174609_m1	TFRC	NM_003234	79	Reference
Hs00430290_m1	UBB	NM_018955	120	Reference
Hs01630490_s1	RPL7A	BX641050	84	Reference
Hs00817975_g1	RPS11	NM_001015	168	Reference
Hs01922548_s1	RPS23	NM_001025	90	Reference
Hs00185390_m1	BAG1	NM_004323	58	
Hs00154355_m1	CD68	NM_001251	68	
Hs01383449_s1	GSTM1	AY532925	65	
(own design)	IGBP5_short	NM_000599	60	Test
(own design)	IGBP5_medium	NM_000599	109	Test
(own design)	IGBP5_long	NM_000599	147	Test

### Data processing and determination of breast cancer classification scores

All the measured cycle threshold (Ct) values represent log_2 _expression levels. These values need to be normalized such that they are comparable across samples and suitable for generating scores. For a gene, a large Ct value corresponds to a low expression level, so the first processing step needed was to reverse the sense of this relationship by letting

Ct' = max(cut_off - Ct, 0)

be the new value for each measured gene. The cut off value was set empirically to 35.0 as any higher raw Ct value was deemed unreliable. This cut off was fixed a priori and kept constant throughout all the experiments reported here. Then, the final value of each target gene was taken to be

ΔCt = max_val*(Ct' - R + cut_off)/(2*cut_off),

where R represents the reference value and was taken as the mean of Ct' values of 5 selected reference genes (GAPDH, GUSB, RPLP0, TFRC, UBB, see Results section for details). The approach guarantees that all ΔCt values are positive and upper bounded by max_val (set to 33 for all the results reported here).

We used the scores associated with three of the gene groups listed in Table 1: the ER, HER2 and Proliferation group. While for the HER2 and Proliferation groups the scores were taken as the average ΔCt value of the genes in the group, for the ER group more weight was given to the ESR1 gene:

ER_score = 0.55*ESR1 + 0.15*(BCL2 + CEGP1 + PGR)

where the gene symbols stand for the corresponding ΔCt values.

Finally, for each tumor a Total score was computed as

Total_score = (Proliferation_score + HER2_score - ER_score + max_val)/3

The Total score, together with the group scores as computed above, are used in all subsequent discussions.

## Results

### Isolation of RNA from FFPE material

**Figure 2:**
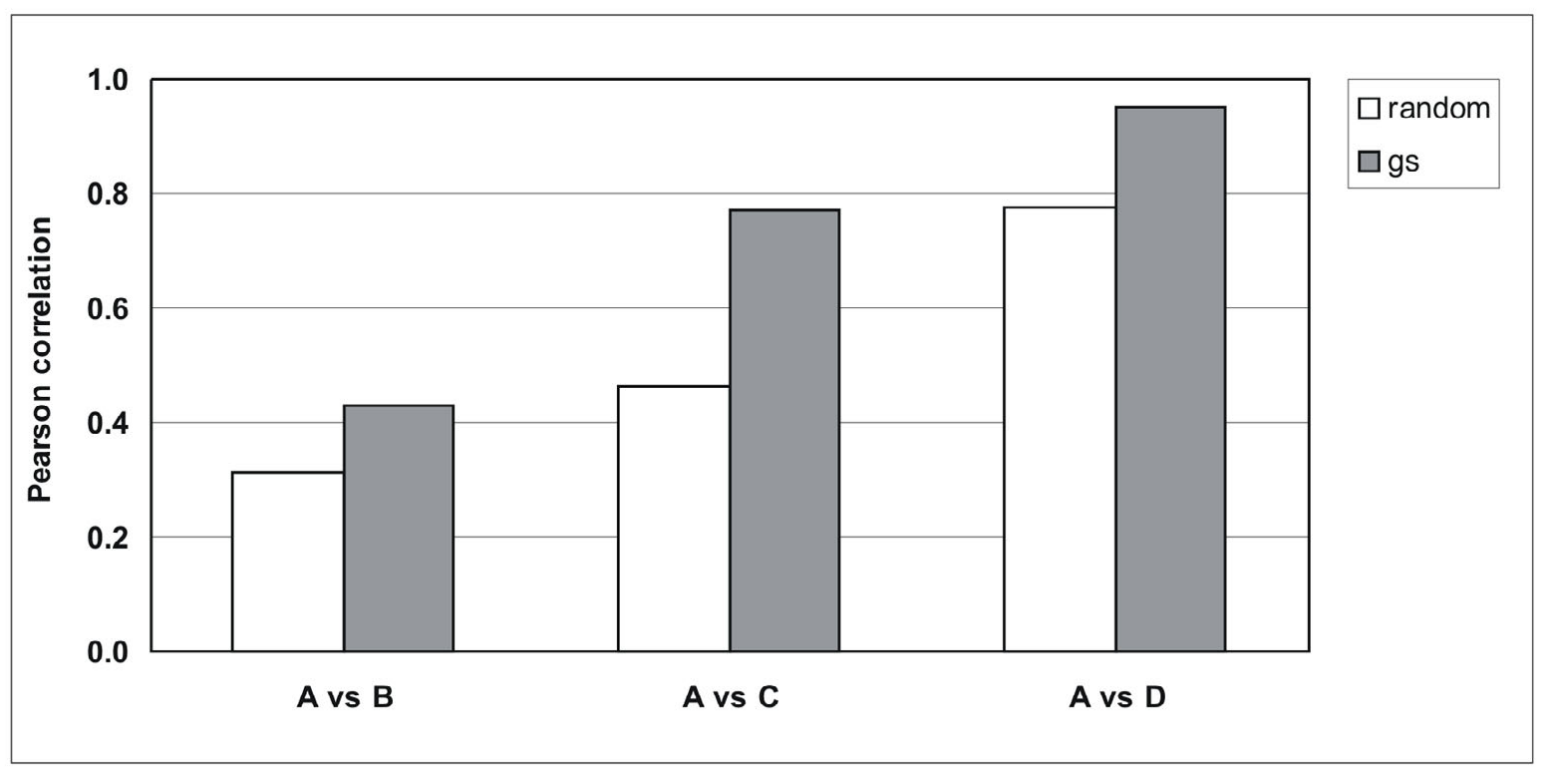
**Comparison of RNAs isolated according to different protocols**. RNA was reverse transcribed in the presence of random primers (white boxes) or gene-specific primers (hatched boxes). Gene expression was measured from an equivalent of 4 ng of RNA by QPCR for five reference genes (GAPDH, GUSB, RPLP0, TFRC and UBB). Pearson correlations were computed between matched Cts for the five reference genes and each tumor RNA isolated from FF (A) and FFPE material. Shown are correlations between intact RNA and RNA isolated from FFPE material according to the RNeasy FFPE protocol (A versus B), intact RNA and RNA isolated from FFPE material according to the ncLysis system (A versus C) and intact RNA and RNA isolated according to our own protocol (A versus D).

Total RNA was isolated from FF human breast cancer specimen which resulted in intact RNA in all samples (Fig. 1, lanes A, shown are RNAs from two representative tumors from a series of 14 tumors). RNA from FF tissue was used as reference for partially fragmented RNA isolated from FFPE material of the same tumors. RNA was assessed by capillary electrophoresis. The size distribution of RNA isolated according to our own protocol was in the range of 200 to 1000 nucleotides (Fig. 1, panel D) while the majority of RNA fragments was in the range of 100 nucleotides when RNA was isolated according to RNeasy FFPE (panel B) or the ncLysis system (panel C). Gene expression was measured by QPCR using 25 commercially available and three own TaqMan assays [13] (Tab. 1). The cycle threshold values (Ct values) were determined from RNAs isolated according to one of the three protocols for FFPE material and compared to Cts obtained with intact RNA of the same tumors. Fig. 2 shows correlation coefficients between intact RNA (A) and FFPE-derived RNAs isolated according to the RNeasy FFPE protocol, (A vs B); the ncLysis system (A vs C); or our own protocol (A vs D) for all 14 tumors using the expression levels of 5 genes (GAPDH, GUSB, RPLP0, TFRC, UBB; see below). The cDNAs were made in the presence of random (white boxes) or gene-specific primers (gray boxes). Clearly, correlation coefficients between intact and partially fragmented RNA were higher with gene-specific primers than random primers and RNA isolated according to our own protocol resulted in cDNA which performed better in QPCR than cDNA made from RNA isolated according to RNeasy FFPE and ncLysis protocols.

### Parameters affecting the RNA quality and QPCR

**Figure 3:**
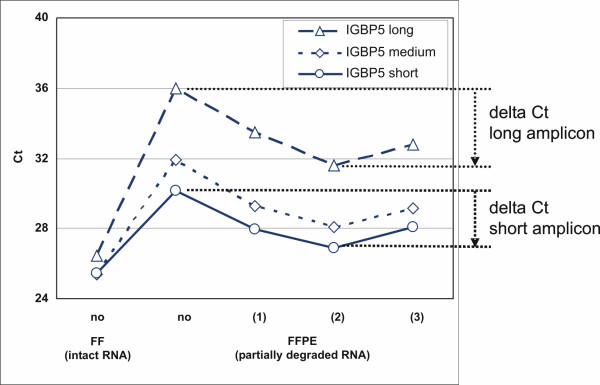
**De-modification of RNA results in higher efficiency during subsequent QPCR**. RNA was isolated from FFPE material according to our own protocol and compared to intact RNA derived of FF tissue. RNA samples were reverse transcribed without previous de-modification (labeled "no") or after de-modification at room temperature (1), 94°C and pH 8.0 (2) or 94°C and pH 5.0 (3). Each RNA was tested by QPCR using three amplicons for IGBP5. Primers used code for short (60 bp, □), medium-size (109 bp, ◇) or long amplicons (147 bp, △). Shown are raw Ct values from intact RNAs from FF material and from RNAs derived of FFPE material of the same tumors. The benefit of de-modification is visualized as delta Ct values. They are indicated for short and long amplicons (dotted lines).

Several parameters were systematically optimized to improve the protocol for RNA isolation from FFPE-derived sections. For example, QPCR made in the presence of primers specific for large amplicons (Fig. 3, dashed line) is very sensitive to RNA fragmentation and modification resulting in higher Ct values than primers specific for medium-size amplicons (dotted line) or short amplicons (non-interrupted line). In addition, the effect of de-modification of FFPE-derived RNA is apparent: the Ct determined from de-modified RNA is 3 or more units lower than the Ct measured from the same RNA but without de-modification. The effect was consistently observed with several tumors and also when expression was measured with TaqMan assays from Applied Biosystems (data not shown). The optimum time of demodification was 20 min, longer times led to higher Ct values (not shown).

**Figure 4:**
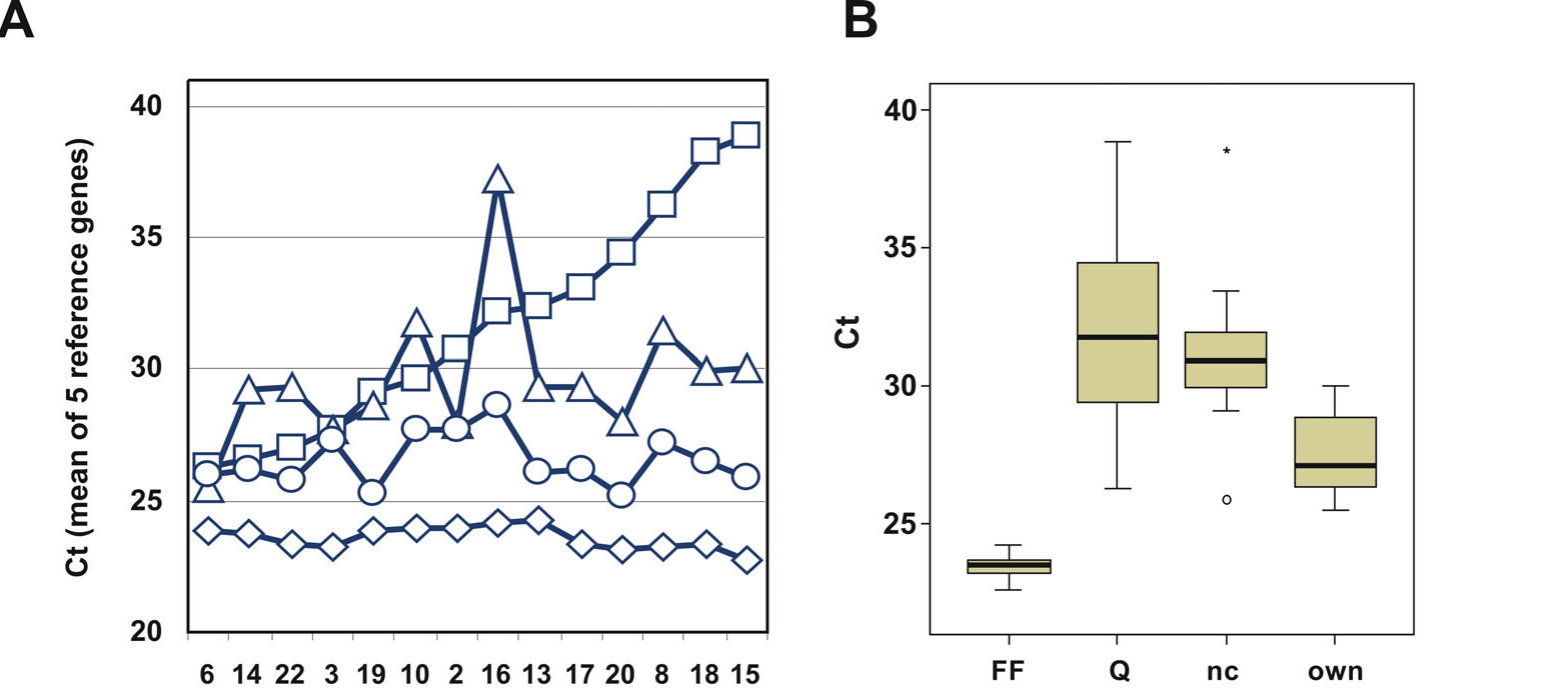
**Comparison of RNA isolation methods**. Shown are the means of raw Cts of five reference genes (GAPDH, GUSB, RPLP0, TFRC, UBB) for intact RNA (◇, FF) and for RNA isolated from matched FFPE material according to the protocols of Qiagen (□, Q), Applied Biosystems (△, AB) and our own (○, own). Individual mean Cts of the 14 tumors and summarized box plots of Cts are shown in panel A and panel B, respectively. Tumors are aligned according to increasing Ct in FFPE-derived RNA (Qiagen protocol).

The different protocols of RNA isolation from FFPE material were further compared by measuring expression levels of reference genes in the 14 tumors and by comparing the results to Cts generated from corresponding intact RNAs (Fig. 4). Experimental variation was reduced by comparing mean Ct values from 5 reference genes (GAPDH, GUSB, RPLP0, TFRC, UBB) instead of their single values. Mean Cts of the five reference genes were plotted for each tumor and each protocol (panel A) and their distribution summarized (panel B). As expected, the Ct values generated with intact RNA resulted in the lowest and most stable Cts (diamonds). RNA prepared from FFPE tissue according to our own protocol (circles) resulted in higher but fairly constant Ct values (compare diamonds and circles). RNA isolated according to the RNeasy FFPE protocol (squares) and the ncLysis protocol (triangles) resulted in Ct values that were not only much higher than with intact RNA, they also exhibited large variations among different isolates when compared to corresponding Cts based on intact RNA. This result suggests a generally poorer and more variable quality of RNA isolated according to the two commercial protocols than our own protocol, leading to relatively large variations of Cts for the 5 reference genes among the different tumors. The Ct values generated from RNA isolated according to our own protocol were on average 2.9 units higher than Cts from intact RNA. RNA isolated according to RNeasy FFPE and ncLysis were 7.6 and 5.8 units higher than Cts from intact RNA of the same tumors, respectively (Fig. 4B). Standard deviations of Cts for the 14 tumors were 0.45 for intact RNA, and 4.21, 2.69 and 1.01 for FFPE-derived RNA isolated according to the RNeasy FFPE, ncLysis and our own protocol, respectively.

**Figure 5:**
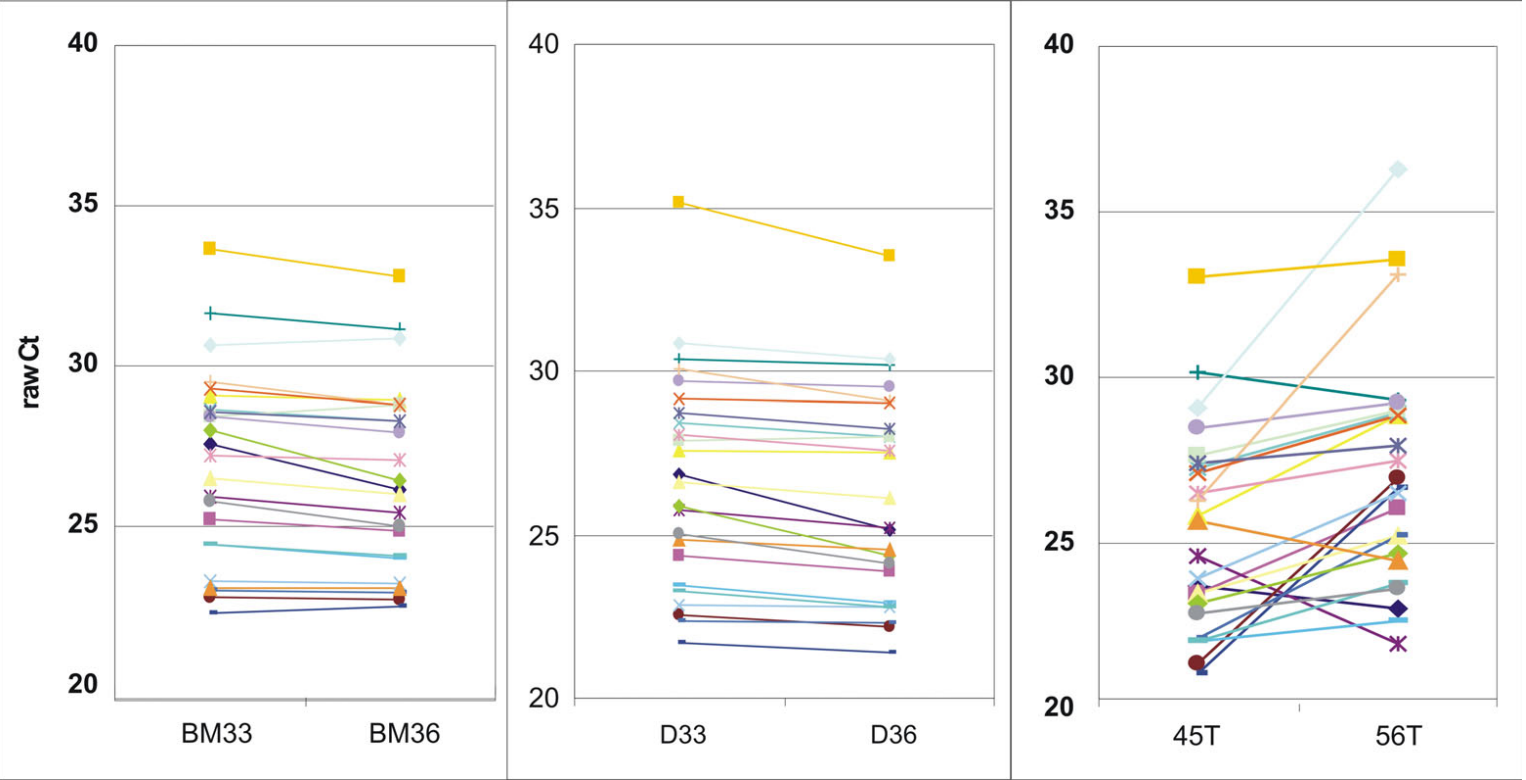
**Reproducibility of RNA isolation from FFPE material**. The RNAs were isolated from paraffin blocks according to our own protocol. BM33 and BM36 (panel A) are two separate RNAs isolated from tissue block "BM", D33 and D36 are RNAs isolated from block "D" (panel B). For comparison, 45T and 56T originate from two distinct tumors isolated from one patient (panel C). Gene expression was measured by QPCR for 24 genes and raw Ct values are shown for each gene measured from the two matching RNAs.

An important aspect when working with RNA from FFPE material relates to the reproducibility of the RNA isolation procedure. This was directly tested for our own protocol by isolating independent samples of RNA from closely matched FFPE sections of the same tissue block and measuring gene expression by QPCR from both RNAs (Fig. 5A and 5B showing two representative examples). RNAs were also isolated from two independent tumors from the same patient, resulting in a third panel of data sets (C). Data points are shown as polygonal diagrams of raw Cts for each gene measured. Horizontal, parallel lines indicate closely similar expression, crossing lines indicate discrepancies between two measurements in matched samples. The Pearson correlation of raw Cts between matched samples was 0.99 for replicates shown in panels A and B and 0.74 for results shown in panel C.

### Normalization

**Figure 6:**
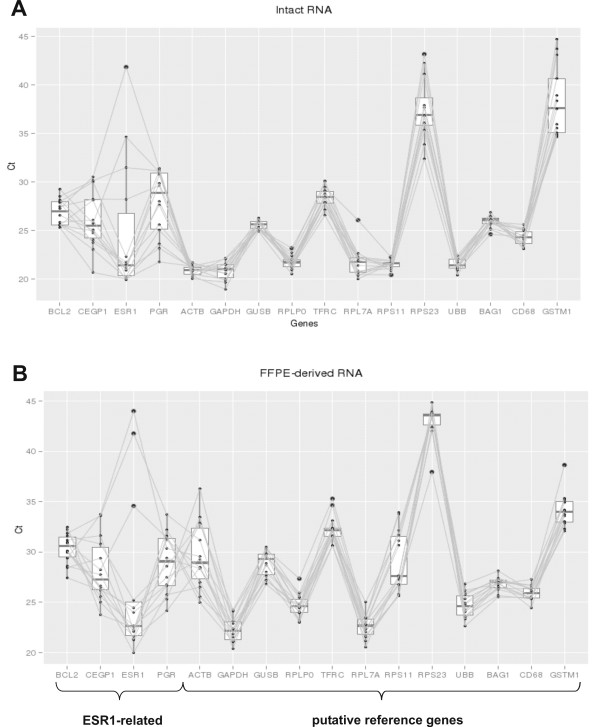
**Stability of reference gene expression in RNA isolated from FF and FFPE material**. Raw Cts are shown for 9 putative reference genes (ACTB, GAPDH, GUSB, RPLP0, TFRC, RPL7A, RPS11, RPS23 and UBB). Results based on intact RNA derived of FF material (A) and based on RNA isolated according to our own protocol from FFPE material (B) are depicted for all the 14 tumors. The Ct values for 4 ER-related genes (BCL2, CEPG1, ESR1 and PGR) are shown for comparison (left).

Results generated in the presence of partially fragmented RNA cannot be directly aligned with results produced from intact RNA and a suitable normalization is required to eliminate or reduce the effects of fragmentation and residual modification in RNA from FFPE material. Nine putative reference genes were selected from the literature [14] and from microarray results [15]. Expression was measured from intact and FFPE-derived RNA and raw Cts from all the 14 tumors are plotted for each putative reference gene (Fig. 6). Analyses based on intact RNAs revealed that 8 of the 9 tested genes performed similarly well (panel A). RPS23 which was hardly measurable (mean Ct in intact RNA > 37) was characterized by a large variation between the different tumors. A slightly higher variation was observed when expression levels were compared for FFPE-derived RNAs (panel B): GAPDH, GUSB, RPLP0, TFRC, RPL7A and UBB showed a similar performance and small variations between the 14 tumors as was seen with intact RNA. In contrast, the Ct values with RNA from FFPE material revealed larger variations for ACTB and RPS11 and therefore, the two genes were excluded as reference genes. The ACTB and RPS11 amplicons are larger than amplicons for the other reference genes and also for the test genes (Tab. 1, see also Fig. 3). Five genes were used as reference genes: GAPDH, GUSB, RPLP0, TFRC and UBB. For comparison, raw Ct values are shown for 4 genes related to the ER response (BCL2, CEPG1, ESR1, PGR) (Fig. 6, left). As expected, a high variation was observed for these genes between the 14 tumors. Protocols B and C did not yield enough usable data, precluding the data from further analysis. For example, protocol B did not have data for all the reference genes and for protocol C several test genes could not be measured reliably (e.g. BCL2, PGR of the ER group).

RNAs isolated from FFPE material according to our own protocol were also compared to RNA derived of kryo-preserved material of the same tumors in a different way. The arithmetic mean of the five reference genes (GAPDH, GUSB, RPLP0, TFRC and UBB) was used for normalizing expression values of all the genes in each RNA. Normalized expression values were compared between intact and FFPE-derived RNA for each gene and each tumor [see Additional File 1]. Good conservation of inter-tumor differences were observed between kryo-preserved and FFPE samples for most genes.

### Module scores

**Figure 7:**
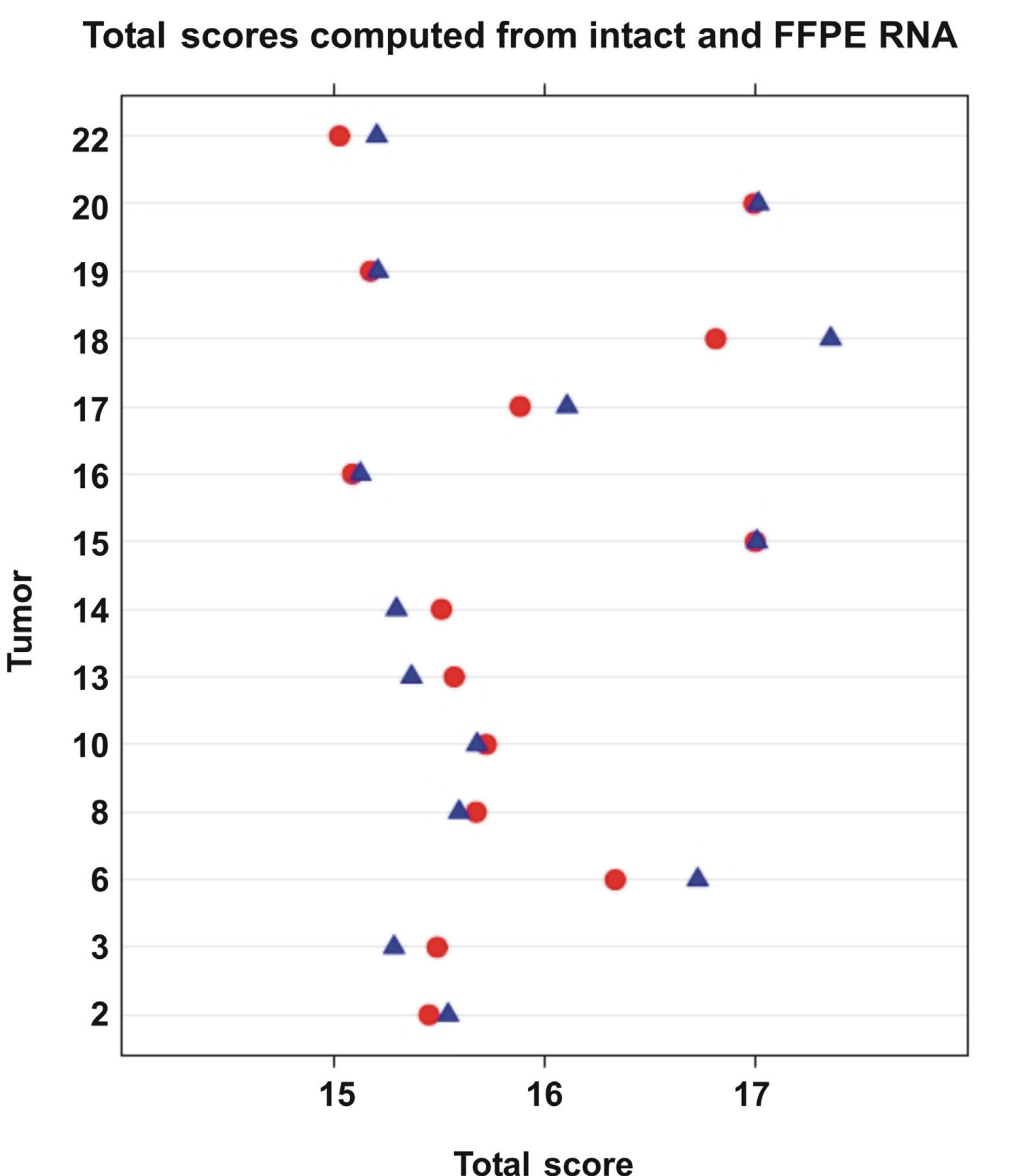
**Comparison of Total scores computed from intact and FFPE-derived RNA**. Total scores were computed from normalized expression values based on the results of intact RNA (○) and FFPE-derived RNA (△, own protocol) as described in the Methods section. They are shown separately for each of the 14 tumors.

**Figure 8:**
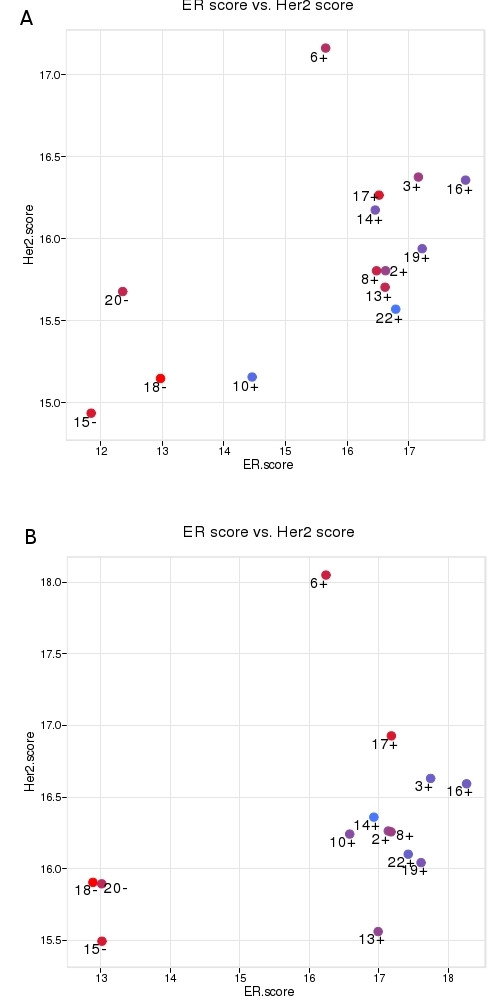
**Module scores**. ER, HER2 and proliferation scores were computed from expression values of 14 breast cancers and visualized in a scatter plot. The ER score was determined from four genes, the HER2 score from 2 and the proliferation score from 5 genes (see Methods). Tumors are positioned according to their ER score (x-axis) and HER2 score (y-axis). Proliferation scores are color coded. The histological ER status is indicated by a "-" or "+" sign next to the tumor numbers in the plot. The results were computed from intact RNA derived of FF material (A) and RNA isolated from FFPE material according to our own protocol (B). Individual scores for each tumor are given in Table 2.

Normalized expression values were also used to compute scores representing ER-related genes (ESR1, PGR, BCL2, CEPG1), HER2-related genes (HER2 and GRB7), genes related to proliferation (STK15/AURKA, CCNB1, MYBL2, MKI67, BIRC5/SURV) and a Total score representing all the genes of the three scores (for details see Methods). The computation of biologically meaningful scores with multiple genes instead of relying on just one has the scope to reduce noise variation. Module scores and Total scores were computed separately from normalized expression values of intact RNAs (circles) and of RNAs isolated according to our own protocol (triangles) and Total scores are depicted separately for each tumor (Fig. 7). The figure demonstrates that similar values are obtained for each tumor irrespective of whether they are computed from intact RNA or from RNA derived of FFPE material. This suggests that scores can be computed with RNA from FF samples as well as with RNA from FFPE samples. ER and HER2 scores were visualized in scatter plots, where the ER and HER2 scores were represented on the x- and y-axis, respectively (Fig. 8A and 8B). It was apparent that the three immunohistochemically ER-negative tumors have low ER scores (#15, #18, #20) and the only immunohistochemically HER2 positive tumor (#6) among the 14 tested tumors had a high HER2 score and an intermediate ER score (see also Table 2). The remaining tumors were all ER positive as assessed by immunohistochemistry (IHC) and they had relatively high ER scores. ER-negative and HER2-positive tumors all had high Proliferation scores (visualized by the red color of the dots). A larger spectrum of Proliferation scores (from blue to red) was found for ER positive tumors. Similar distributions were found when scores were computed from intact RNA (Fig. 8A) and FFPE-derived RNA that was isolated according to our own protocol (B). A different presentation of scores is shown where ER, HER2, Proliferation and Total Scores are plotted separately for each tumor [see Additional file 2]. The scores determined from the 14 FF and FFPE-derived samples are in the same range and only few tumors were classified in a different order between intact and FFPE-derived RNAs (leading to crossing lines).

**Table 2:** Clinical and molecular parameters of breast cancers. Clinical and molecular parameters are given for each breast cancer used in this study. Module scores for each tumor were calculated from the results based on intact RNA (FF material) and based on RNA isolated from FFPE material according to our own method. N.A., data not available.

	**Clinical classification**			**Immunohistochemistry**			**Module Score (FF/FFPE)**			
		
**Tu#**	**T**	**N**	**Grade**	**ER**	**PR**	**ErbB2**	**ER**	**HER2**	**Prolif.**	**Histological type**
2	2	0	3	70% pos.	neg.	1+	16.6/17.1	15.8/16.3	14.2/14.5	invasive ductal
3	2	1a	2	70% pos.	pos.	1+	17.2/17.7	16.4/16.6	14.3/14.0	mixed (duct./lob)
6	1c	3a	3	>90% pos.	pos.	3+	15.7/16.2	17.2/18.1	14.5/15.4	invasive ductal
8	2	2a	3	>90% pos.	pos.	2+	16.5/17.2	15.8/16.3	14.7/14.7	invasive ductal
10	1c	N.A.	2	>90% pos.	pos.	2+	14.5/16.6	15.2/16.2	13.5/14.4	invasive ductal
13	2	3a	3	>90% pos.	neg.	1+	16.6/17.0	15.7/15.6	14.6/14.5	invasive ductal
14	1c	N.A.	2	>90% pos.	neg.	1+	16.5/16.9	16.2/16.4	13.8/13.5	invasive ductal
15		N.A.	3	neg.	neg.	0	11.8/13.0	14.9/15.5	14.9/15.5	invasive ductal
16	2	N.A.	1	65% pos.	pos.	0	17.9/18.3	16.4/16.6	13.8/14.1	invasive ductal/cribriform
17	2	0	3	>90% pos.	pos.	2+	16.5/17.2	16.3/16.9	14.9/15.6	invasive ductal
18	2	N.A.	3	neg.	neg.	0	13.0/12.9	15.1/15.9	15.2/16.0	invasive ductal
19	2	N.A.	2	>90% pos.	pos.	0	17.2/17.6	15.9/16.0	13.8/14.2	invasive ductal
20	1c	N.A.	2	neg.	neg.	0	12.4/13.0	15.7/15.9	14.6/15.2	invasive ductal
22	2	0	2	N.A.	N.A.	N.A.	16.8/17.4	15.6/16.1	13.3/13.9	invasive ductal

The similarity between the results generated from intact and partially fragmented RNA was also assessed by calculating Pearson correlation coefficients between the scores of both RNAs. Correlation coefficients (and corresponding p-values and 95% confidence intervals) were 0.966 (p = 2.071*10^-8^, CI = 0.893; 0.989), 0.856 (p = 9.32*10^-5^, CI = 0.597; 0.954) and 0.833 (p = 2.177*10^-4^, CI = 0.541; 0.946) for ER, HER2 and Proliferation scores, respectively. The corresponding Spearman correlations were 0.938 (p < 2.2*10^-16^), 0.851 (p = 1.167*10^-4^) and 0.867 (p = 2.048*10^-5^), respectively.

## Discussion

Methods and protocols for RNA isolation from formalin-fixed tissues have been published since almost 20 years [16-32].

RNA was quantified by dot blot hybridization [23], semi-quantitative PCR [19] and more recently, by QPCR [24,18,26,13,17,33,32] and other methods [28-30]. RNA derived of FFPE material is not only partially hydrolyzed but also chemically modified: formalin reacts with nucleotides leading to the formation of methylol groups in nucleobases. These groups tend to further react and form intra- and inter-molecular methylene bridges in RNA, DNA [34,35,31] and protein [36]. As a result, reverse transcription is impaired and threshold cycle values (Ct values) increase during subsequent QPCR.

The protocol for RNA isolation described here was complemented by adding a separate demodification step which involves incubation at elevated temperature in a buffer containing ammonium chloride which favors the reversion of methylol groups to amino groups in nucleobases. It does not only improve the efficiency of downstream applications (mainly reverse transcription), it also improves the recovery of RNA from FFPE sections. RNA yield and quality can be further improved by extensive digestion of FFPE material with protease in a buffer containing guanidinium thiocyanate. Reverse transcription in the presence of gene-specific primers prevents the initiation of cDNA synthesis inside amplicons and therefore, cDNA made in the presence of gene-specific primers is a better template for QPCR than cDNA made from random primers (Fig. 2). Several papers have demonstrated that QPCR with primers coding for short amplicons are more efficient than primers coding for long amplicons [17,20,24,13,32].

Finally, normalization of raw data is used to eliminate or at least reduce the effect of poorer quality of starting RNA. Various approaches of normalization were proposed in the literature [37,14,38,32]. They are based on calculating relative expression values: expression levels of genes of interest are expressed relative to the expression of one or a panel of several suitable reference genes. An ideal reference gene has a stable expression level in all the samples under investigation. As such "ideal" reference gene normally does not exist, the mean or median expression level of several suitably chosen reference genes is used as a relatively stable reference Ct value. We used a formalized approach to characterize all candidate reference genes. Candidate reference genes were ranked according to their standard deviations of raw Ct values in RNA from FF and FFPE material. The final rank of each candidate reference gene was taken as the mean of the two ranks obtained with RNA from intact and FFPE material. Genes with higher ranks were excluded as reference genes.

We also applied GeNorm [14] to characterize candidate reference genes: ACTB and RPS11 had poorest stability measure M [14] for FFPE-derived RNA and RPL7A had a poor stability measure when RNA from FF material was tested (data not shown). For these reasons GAPDH, GUSB, RPLP0, TFRC and UBB were used as reference genes in this study.

Our own RNA isolation protocol was compared to RNA that was isolated from the same material but according to commercial protocols and products (Qiagen RNeasy FFPE and ncLysis system of Applied Biosystems). Additional products for FFPE material from commercial providers (e.g. Stratagene, Ambion) were tested and the results obtained with our own protocol were superior to all tested commercial products (data not shown).

We determined module scores for each of the 14 tumors in this study. The limited number of samples does not allow statements about the clinical significance of module scores but they can be used to compare scores computed from intact RNA from FF material and RNA isolated from FFPE according to our own protocol. Pearson correlations between these RNAs in the 14 tumors were 0.966, 0.856 and 0.833 for ER, HER2 and Proliferation scores, respectively. As kryo-preserved RNA and RNA from FFPE material always originated from different portions of the same tumor, a certain variation of gene expression cannot be excluded and, as a consequence, part of the observed variability between kryo and FFPE material may be attributed to biological heterogeneity in the tumors. The three module scores were combined to a Total score. The Total score is similar to the recurrence score described by Paik [11], with high expression of genes related to proliferation and HER2 and low expression of ER-related genes indicating higher risk.

The data generated from FF and FFPE material were also compared to ER and HER2 levels assessed by IHC results from the same tumors. Three tumors (#15, #18, #20) were ER-negative and one was strongly HER2-positive (#6) (Tab. 2). The same tumors had low ER scores when assessed by QPCR (Fig. 8). Tumor #6 had a high HER2 score and an intermediate ER score. These results are in good agreement with the expected distribution of the three scores [15,39]. By comparing QPCR based data with well known tumor subtypes allowed to validate the protocols developed here, even if no new biological findings are provided. The primary issue of this work was to document that stable and robust expression values can be determined from FFPE-derived RNA which are close to the values computed from intact RNA of the same tumors. The optimization and validation of the scoring procedure remains an important issue but obviously, the available number of samples is not sufficient to deal with this aspect and it will be addressed separately and on a larger collection of samples.

While IHC results are at most semi-quantitative, QPCR-based results reflect more accurately the expression level of genes in question. The module scores proposed here integrate quantitative gene expression data from several genes, this makes the resulting scores more robust than measurements based on single genes. QPCR is not only quantitative, it is also very sensitive over a large dynamic range. The number of genes which can be measured by QPCR is not limited and additional genes and module scores can be included in the analysis if this will be required.

Importantly, certain predictive parameters still cannot be determined with current technologies. For example, breast cancers are classified into histological grade 1, 2 or 3. This grading most likely reflects the proliferative state of tumor cells [40]. Grading may be especially important as high grade tumors seem to respond more favorably to chemotherapy than low grade tumors. Unfortunately, many tumors are histological grade 2 and for those tumors the benefit is not clear. Paik and co-workers documented that their recurrence score (RS) was also predictive for a response to chemotherapy [41]. The RS defined by Paik and coworkers is composed of 16 test genes mainly representing ER response genes, proliferation-associated genes, HER2-related genes and invasion genes and 5 genes for normalization [11,41].

The genes selected for this study (Tab. 1) were selected from published DNA chip studies with breast cancer samples [15]. They mostly coincide with the genes used by Paik ad co-workers.

## Conclusion

The results presented in this study reveal that RNA isolated from FFPE material according to the protocol developed in our laboratory can be used for expression measurements by QPCR although the RNA is partially degraded. The optimized isolation and de-modification procedures combined with a normalization procedure results in stable and robust gene expression data. Robustness of results was further increased by computing scores from several genes representing the hormonal and the proliferation status of the tumor. Molecular profiling from FFPE material may be of interest for routine diagnostics in the near future as FFPE material is always available [42]. Similarly, molecular profiling from FFPE material may be of great interest in the context of existing and newly planed clinical trials for which only formalin-fixed samples exist.

## Abbreviations

ER, estrogen receptor; FF, fresh frozen tissue; FFPE, formalin-fixed, paraffin-embedded tissue; IHC, immunohistochemistry; PGR, progesterone receptor; QPCR, quantitative polymerase chain reaction.

## Competing interests

The author(s) declare that they have no competing interests.

## Authors' contributions

AO and AB developed the procedure, performed validation studies and were involved in all the experiments presented here. HJA and SA contributed clinical and pathological information. JA and SM participated in the design of the study and VP and MD performed the statistical analysis and developed the scoring system. RJ conceived the study, participated in its design and coordination and drafted the manuscript. All the authors read and approved the final manuscript.

## Pre-publication history

The pre-publication history for this paper can be accessed here:



## Supplementary Material

Additional file 1 **Comparison of normalized expression for each gene in FF and FFPE material**. Expression was determined by QPCR from RNA derived of FF and FFPE material (own protocol). Normalized expression levels (see Methods for details) are shown for each gene and the 14 tumors as polygonal plots. Click here for file

Additional file 2 **Polygonal representation of ER, HER2, Proliferation and Total scores**. Gene expression was measured from RNA derived of FF and FFPE material (own protocol) and ER, HER2, proliferation and Total scores were computed for each RNA of the 14 tumors and results are shown as polygonal plots. Parallel lines indicate good correlations and crossing lines are indicative for discrepancies between scores computed from FF and FFPE-derived RNA Click here for file

## References

[B1] Sorlie T, Perou CM, Tibshirani R, Aas T, Geisler S, Johnsen H, Hastie T, Eisen MB, van de Rijn M, Jeffrey SS, Thorsen T, Quist H, Matese JC, Brown PO, Botstein D, Eystein Lonning P, Borresen-Dale AL (2001). Gene expression patterns of breast carcinomas distinguish tumor subclasses with clinical implications. Proc Natl Acad Sci U S A.

[B2] Sorlie T, Tibshirani R, Parker J, Hastie T, Marron JS, Nobel A, Deng S, Johnsen H, Pesich R, Geisler S, Demeter J, Perou CM, Lonning PE, Brown PO, Borresen-Dale AL, Botstein D (2003). Repeated observation of breast tumor subtypes in independent gene expression data sets. Proc Natl Acad Sci U S A.

[B3] Sotiriou C, Neo SY, McShane LM, Korn EL, Long PM, Jazaeri A, Martiat P, Fox SB, Harris AL, Liu ET (2003). Breast cancer classification and prognosis based on gene expression profiles from a population-based study. Proc Natl Acad Sci U S A.

[B4] Perou CM, Sorlie T, Eisen MB, van de Rijn M, Jeffrey SS, Rees CA, Pollack JR, Ross DT, Johnsen H, Akslen LA, Fluge O, Pergamenschikov A, Williams C, Zhu SX, Lonning PE, Borresen-Dale AL, Brown PO, Botstein D (2000). Molecular portraits of human breast tumours. Nature.

[B5] Rampaul RS, Pinder SE, Elston CW, Ellis IO (2001). Prognostic and predictive factors in primary breast cancer and their role in patient management: The Nottingham Breast Team. Eur J Surg Oncol.

[B6] Goldhirsch A, Glick JH, Gelber RD, Coates AS, Thurlimann B, Senn HJ (2005). Meeting highlights: international expert consensus on the primary therapy of early breast cancer 2005. Ann Oncol.

[B7] Sotiriou C, Powles TJ, Dowsett M, Jazaeri AA, Feldman AL, Assersohn L, Gadisetti C, Libutti SK, Liu ET (2002). Gene expression profiles derived from fine needle aspiration correlate with response to systemic chemotherapy in breast cancer. Breast Cancer Res.

[B8] van 't Veer LJ, Dai H, van de Vijver MJ, He YD, Hart AA, Mao M, Peterse HL, van der Kooy K, Marton MJ, Witteveen AT, Schreiber GJ, Kerkhoven RM, Roberts C, Linsley PS, Bernards R, Friend SH (2002). Gene expression profiling predicts clinical outcome of breast cancer. Nature.

[B9] Shi L, Reid LH, Jones WD, Shippy R, Warrington JA, Baker SC, Collins PJ, de Longueville F, Kawasaki ES, Lee KY, Luo Y, Sun YA, Willey JC, al. (2006). The MicroArray Quality Control (MAQC) project shows inter- and intraplatform reproducibility of gene expression measurements. Nat Biotechnol.

[B10] Loi S, Haibe-Kains B, Desmedt C, Lallemand F, Tutt AM, Gillet C, Ellis P, Harris A, Bergh J, Foekens JA, Klijn JG, Larsimont D, Buyse M, Bontempi G, Delorenzi M, Piccart MJ, Sotiriou C (2007). Definition of clinically distinct molecular subtypes in estrogen receptor-positive breast carcinomas through genomic grade. J Clin Oncol.

[B11] Paik S (2006). Molecular profiling of breast cancer. Curr Opin Obstet Gynecol.

[B12] Cronin M, Pho M, Dutta D, Stephans JC, Shak S, Kiefer MC, Esteban JM, Baker JB (2004). Measurement of gene expression in archival paraffin-embedded tissues: development and performance of a 92-gene reverse transcriptase-polymerase chain reaction assay. Am J Pathol.

[B13] Antonov J, Goldstein DR, Oberli A, Baltzer A, Pirotta M, Fleischmann A, Altermatt HJ, Jaggi R (2005). Reliable gene expression measurements from degraded RNA by quantitative real-time PCR depend on short amplicons and a proper normalization. Lab Invest.

[B14] Vandesompele J, De Preter K, Pattyn F, Poppe B, Van Roy N, De Paepe A, Speleman F (2002). Accurate normalization of real-time quantitative RT-PCR data by geometric averaging of multiple internal control genes. Genome Biol.

[B15] Wirapati P, Kunkel S, Goldstein DG, Farmer P, Pradervand S, Haibe-Kains B, Desmedt C, Sengstag T, Schütz F, Piccart M, Sotiriou C, Delorenzi M (2007). Integrative analysis of gene-expression profiles: toward a unified understanding of breast cancer subtyping and prognosis signatures.

[B16] Shibutani M, Uneyama C, Miyazaki K, Toyoda K, Hirose M (2000). Methacarn fixation: a novel tool for analysis of gene expressions in paraffin-embedded tissue specimens. Lab Invest.

[B17] Abrahamsen HN, Steiniche T, Nexo E, Hamilton-Dutoit SJ, Sorensen BS (2003). Towards quantitative mRNA analysis in paraffin-embedded tissues using real-time reverse transcriptase-polymerase chain reaction: a methodological study on lymph nodes from melanoma patients. J Mol Diagn.

[B18] Godfrey TE, Kim SH, Chavira M, Ruff DW, Warren RS, Gray JW, Jensen RH (2000). Quantitative mRNA expression analysis from formalin-fixed, paraffin-embedded tissues using 5' nuclease quantitative reverse transcription-polymerase chain reaction. J Mol Diagn.

[B19] Houze TA, Gustavsson B (1996). Sonification as a means of enhancing the detection of gene expression levels from formalin-fixed, paraffin-embedded biopsies. Biotechniques.

[B20] Koopmans M, Monroe SS, Coffield LM, Zaki SR (1993). Optimization of extraction and PCR amplification of RNA extracts from paraffin-embedded tissue in different fixatives. J Virol Methods.

[B21] Lewis F, Maughan NJ, Smith V, Hillan K, Quirke P (2001). Unlocking the archive--gene expression in paraffin-embedded tissue. J Pathol.

[B22] Reichmuth C, Markus MA, Hillemanns M, Atkinson MJ, Unni KK, Saretzki G, Hofler H (1996). The diagnostic potential of the chromosome translocation t(2;13) in rhabdomyosarcoma: a Pcr study of fresh-frozen and paraffin-embedded tumour samples. J Pathol.

[B23] Rupp GM, Locker J (1988). Purification and analysis of RNA from paraffin-embedded tissues. Biotechniques.

[B24] Specht K, Richter T, Muller U, Walch A, Werner M, Hofler H (2001). Quantitative gene expression analysis in microdissected archival formalin-fixed and paraffin-embedded tumor tissue. Am J Pathol.

[B25] Stanta G, Bonin S (1998). RNA quantitative analysis from fixed and paraffin-embedded tissues: membrane hybridization and capillary electrophoresis. Biotechniques.

[B26] Thomazy VA, Luthra R, Uthman MO, Davies PJ, Medeiros LJ (2002). Determination of cyclin D1 and CD20 mRNA levels by real-time quantitative RT-PCR from archival tissue sections of mantle cell lymphoma and other non-Hodgkin's lymphomas. J Mol Diagn.

[B27] Mies C (1994). A simple, rapid method for isolating RNA from paraffin-embedded tissues for reverse transcription-polymerase chain reaction (RT-PCR). J Histochem Cytochem.

[B28] Bibikova M, Talantov D, Chudin E, Yeakley JM, Chen J, Doucet D, Wickham E, Atkins D, Barker D, Chee M, Wang Y, Fan JB (2004). Quantitative gene expression profiling in formalin-fixed, paraffin-embedded tissues using universal bead arrays. Am J Pathol.

[B29] Bibikova M, Chudin E, Arsanjani A, Zhou L, Garcia EW, Modder J, Kostelec M, Barker D, Downs T, Fan JB, Wang-Rodriguez J (2007). Expression signatures that correlated with Gleason score and relapse in prostate cancer. Genomics.

[B30] Haller AC, Kanakapalli D, Walter R, Alhasan S, Eliason JF, Everson RB (2006). Transcriptional profiling of degraded RNA in cryopreserved and fixed tissue samples obtained at autopsy. BMC Clin Pathol.

[B31] Rait VK, Zhang Q, Fabris D, Mason JT, O'Leary TJ (2006). Conversions of formaldehyde-modified 2'-deoxyadenosine 5'-monophosphate in conditions modeling formalin-fixed tissue dehydration. J Histochem Cytochem.

[B32] Koch I, Slotta-Huspenina J, Hollweck R, Anastasov N, Hofler H, Quintanilla-Martinez L, Fend F (2006). Real-time quantitative RT-PCR shows variable, assay-dependent sensitivity to formalin fixation: implications for direct comparison of transcript levels in paraffin-embedded tissues. Diagn Mol Pathol.

[B33] Hamatani K, Eguchi H, Takahashi K, Koyama K, Mukai M, Ito R, Taga M, Yasui W, Nakachi K (2006). Improved RT-PCR amplification for molecular analyses with long-term preserved formalin-fixed, paraffin-embedded tissue specimens. J Histochem Cytochem.

[B34] Masuda N, Ohnishi T, Kawamoto S, Monden M, Okubo K (1999). Analysis of chemical modification of RNA from formalin-fixed samples and optimization of molecular biology applications for such samples. Nucleic Acids Res.

[B35] Chaw YF, Crane LE, Lange P, Shapiro R (1980). Isolation and identification of cross-links from formaldehyde-treated nucleic acids. Biochemistry.

[B36] Orlando V, Strutt H, Paro R (1997). Analysis of chromatin structure by in vivo formaldehyde cross-linking. Methods.

[B37] Fleige S, Walf V, Huch S, Prgomet C, Sehm J, Pfaffl MW (2006). Comparison of relative mRNA quantification models and the impact of RNA integrity in quantitative real-time RT-PCR. Biotechnol Lett.

[B38] Andersen CL, Jensen JL, Orntoft TF (2004). Normalization of real-time quantitative reverse transcription-PCR data: a model-based variance estimation approach to identify genes suited for normalization, applied to bladder and colon cancer data sets. Cancer Res.

[B39] Fan C, Oh DS, Wessels L, Weigelt B, Nuyten DS, Nobel AB, van't Veer LJ, Perou CM (2006). Concordance among gene-expression-based predictors for breast cancer. N Engl J Med.

[B40] Sotiriou C, Wirapati P, Loi S, Harris A, Fox S, Smeds J, Nordgren H, Farmer P, Praz V, Haibe-Kains B, Desmedt C, Larsimont D, Cardoso F, Peterse H, Nuyten D, Buyse M, Van de Vijver MJ, Bergh J, Piccart M, Delorenzi M (2006). Gene expression profiling in breast cancer: understanding the molecular basis of histologic grade to improve prognosis. J Natl Cancer Inst.

[B41] Paik S, Tang G, Shak S, Kim C, Baker J, Kim W, Cronin M, Baehner FL, Watson D, Bryant J, Costantino JP, Geyer CE, Wickerham DL, Wolmark N (2006). Gene expression and benefit of chemotherapy in women with node-negative, estrogen receptor-positive breast cancer. J Clin Oncol.

[B42] Sun Y, Goodison S, Li J, Liu L, Farmerie W (2007). Improved breast cancer prognosis through the combination of clinical and genetic markers. Bioinformatics.

