# Supplementary figures and images for: Expression profiling with RNA from formalin-fixed, paraffin-embedded material
Source: BMC Med Genomics. 2008 Apr 19;1:9. doi: 10.1186/1755-8794-1-9 (PMC2359756; doi:10.1186/1755-8794-1-9)

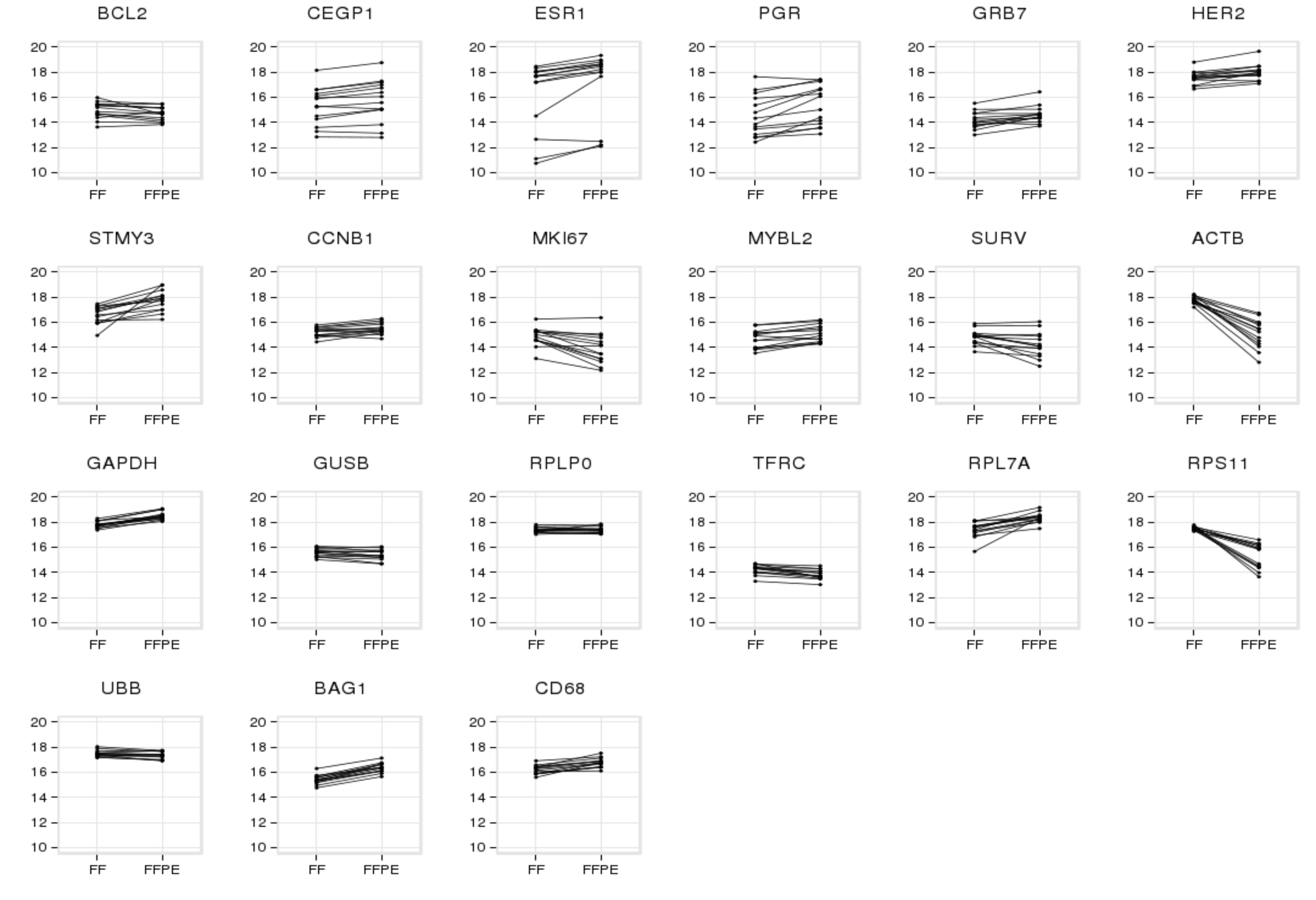

Supplement: Additional file 1 — Comparison of normalized expression for each gene in FF and FFPE material. Expression was determined by QPCR from RNA derived of FF and FFPE material (own protocol). Normalized expression levels (see Methods for details) are shown for each gene and the 14 tumors as polygonal plots. [file 1755-8794-1-9-S1.jpeg]

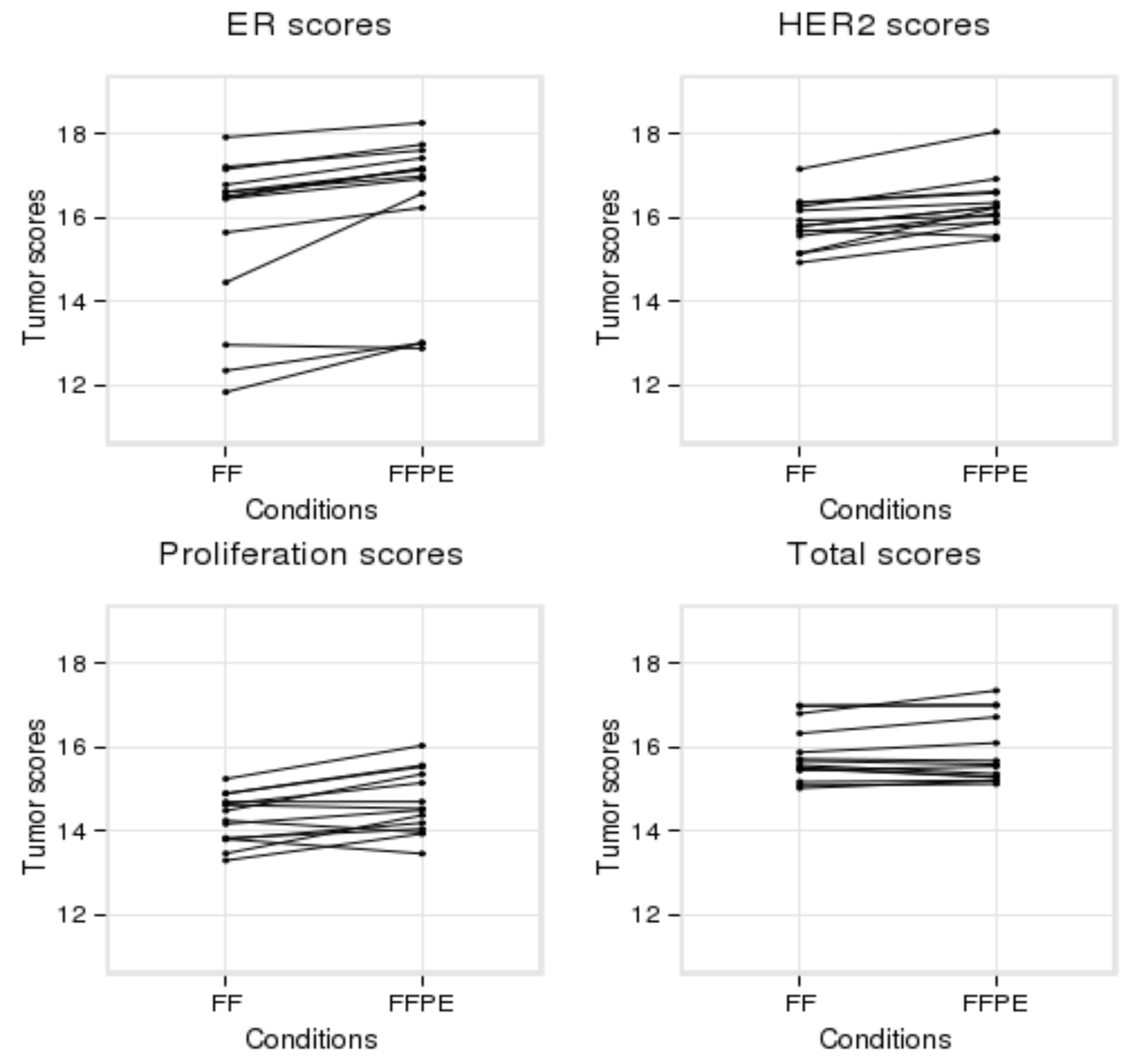

Supplement: Additional file 2 — Polygonal representation of ER, HER2, Proliferation and Total scores. Gene expression was measured from RNA derived of FF and FFPE material (own protocol) and ER, HER2, proliferation and Total scores were computed for each RNA of the 14 tumors and results are shown as polygonal plots. Parallel lines indicate good correlations and crossing lines are indicative for discrepancies between scores computed from FF and FFPE-derived RNA [file 1755-8794-1-9-S2.jpeg]
